# Is *Lycorma delicatula* (Hemiptera: Fulgoridae) a blooming threat to citrus?

**DOI:** 10.1093/jee/toae197

**Published:** 2024-09-11

**Authors:** Marco Molfini, Mari West, Francesc Gómez-Marco, Jorge Braz Torres, Mark Hoddle

**Affiliations:** Department of Entomology, University of California, Riverside, CA, USA; Department of Entomology, University of California, Riverside, CA, USA; Department of Entomology, University of California, Riverside, CA, USA; Sustainable Plant Protection Program, Institut de Recerca i Tecnologia Agroalimentàries, Cabrils, Spain; Departamento de Agronomia-Entomologia, Universidade Federal Rural de Pernambuco, Recife, Brazil; Department of Entomology, University of California, Riverside, CA, USA

**Keywords:** host plant, invasive species, navel orange, nymphal development, survival analysis

## Abstract

Examining the host range of emerging invasive insects is essential to assess their invasion potential and to anticipate the negative impacts of their spread. The ongoing North American invasion of spotted lanternfly (SLF) [*Lycorma delicatula* (White, 1845)] threatens agricultural, urban, and natural areas. The survival and development of SLF nymphs on Washington navel orange [*Citrus sinensis* (L.) Osbeck (Sapindales: Rutaceae)] trees were assessed in a quarantine facility. Results indicated that SLF nymphs can develop to at least the third instar by feeding exclusively on Washington navel orange. This finding suggests that, at least up to the third stage of nymphal development, Washington navel orange might be a suitable host for SLF, highlighting the possibility that this invasive pest represents an unrecognized threat to this globally important crop and possibly to other *Citrus* species.

## Introduction

Spotted lanternfly (SLF), *Lycorma delicatula* (White, 1845), is a generalist planthopper native to parts of East Asia and the Indomalayan region (Bourgoin 2024). This species has attained notoriety as a pest after its accidental introduction into and subsequent spread in the Republic of Korea (2004; Kim and Kim 2005), Japan (2008; Kim et al. 2013) and the United States (2014; Barringer et al. 2015). High-density, rapidly spreading populations pose a significant threat to numerous agricultural commodities, urban shade trees, and native plants (Barringer and Ciafré 2020, Wakie et al. 2020, Huron et al. 2022, Jones et al. 2022).

All motile SLF developmental stages (i.e., 4 nymphal instars and the adult stage) feed exclusively on plant phloem, which causes direct feeding damage and indirect damage through the excretion of copious amounts of honeydew, which promotes sooty mold growth on infested plants (Dara et al. 2015). A highly preferred host for SLF is Tree-of-Heaven (ToH), [*Ailanthus altissima* (Mill.) Swingle (Sapindales: Simaroubaceae)], a globally invasive tree particularly abundant in Europe and North America (Sladonja et al. 2015). Access to ToH significantly enhances the fecundity of female SLF (Uyi et al. 2020, 2021). Additionally, SLF can feed on more than 100 plant species, and feeding preference depends on the plant composition in any given area (Urban 2019, Barringer and Ciafré 2020). SLF reared on a mixed host plant diet exhibit higher fitness and faster development than individuals restricted to a single host plant species (Uyi et al. 2021, Nixon et al. 2022, Elsensohn et al. 2023a).

Due to its generalist ecology and the high availability of ToH, SLF is spreading rapidly within the United States. Moreover, due to numerous invasion bridgeheads in Asia and North America, this pest is an excellent candidate to become a globally invasive species (i.e., paninvasive), with the potential to inflict significant economic and ecological damage in invaded regions (Wakie et al. 2020, Huron et al. 2022, Jones et al. 2022).

Most concerns regarding the spread and establishment of SLF are related to its documented or anticipated damage to important crops, although it may also have negative effects on natural ecosystems (Lee et al. 2019, Nixon et al. 2022) for which data on invasive species are often lacking (Molfini et al. 2020). Major economic damage has been observed in commercial vineyards (*Vitis* spp. L.), and loss of entire productivity in vineyards with severe infestations has been recorded (Urban et al. 2021). While numerous insecticides are effective against SLF (Leach et al. 2019), continuous reinfestations from surrounding areas increase the frequency of insecticide applications, which, in turn, increase associated management costs, environmental damage, and the potential development of insecticide resistance (Urban 2019, Urban et al. 2021, Elmquist et al. 2023).

Considering the high risk of SLF becoming a paninvasive species and its associated negative impacts (Huron et al. 2022), it is crucial to anticipate potential new host species to better assess the future invasive potential of this pest and develop effective plans for early detection and rapid responses (e.g., Barringer and Ciafré 2020, Uyi et al. 2021, Nixon et al. 2022, Elsensohn et al. 2023a, b), including proactive biological control (Gómez Marco et al. 2023).

Plants in the genus *Citrus* L. (Sapindales: Rutaceae) are widely cultivated in over 140 countries throughout the “citrus belt,” which lies approximately within latitudes 40° N and 40° S (Zhong and Nicolosi 2020). Global production of citrus exceeds 124 million tons, and this commodity is in the top quartile of the global market with a trade value of over $15 billion ($US) (Zhong and Nicolosi 2020, OEC.world 2022). To better understand the risk SLF poses to this important economic crop, a quarantine laboratory study was conducted to investigate the suitability of Washington navel orange [*C. sinensis* (L.) Osbeck] plants for the development of SLF nymphs.

## Materials and Methods

Washington navel orange trees were purchased from a local nursery (Riverside, CA) in spring 2022. All experimental plants were maintained at approximately 40 cm height in 6.5 L plastic pots in the University of California, Riverside Insectary and Quarantine Facility (UCR-I&Q) greenhouse until used in experiments. Plants were watered when needed and fertilized with Osmocote (Scotts Miracle-Gro, Marysville, OH) per manufacturer’s instructions. Prior to the start of the experiment, plants were transferred to mesh cages [W60 × H60 × D60 cm (BugDorm-BD2S120 Insect Rearing Cage, MegaView Science Co., Ltd., Taiwan)] in a rearing room maintained at 25 °C and 50% RH with a 16:8 L:D cycle. White plastic poster board was placed around the trunks of trees and on top of pots to prevent falling SLF nymphs from landing in potting soil and to facilitate rapid detection of dead nymphs and exuviae.

SLF egg masses were hand-collected in Millville, NJ, Moorestown, NJ, and Falls Township, PA, from 14 to 16 February 2023. SLF egg masses were removed from the bark of host plants using chisels and hammers and stored under ambient conditions in ventilated plastic containers, separated into layers with paper shop towels (Shop Towels Original, Scott, Philadelphia, PA, USA) to provide moisture control and cushioning from impacts during transportation. All field-collected SLF egg masses were transported to the University of California Riverside Insectary and Quarantine Facility (UCR-I&Q) under USDA-APHIS permit number P526P-21-06935.

**Fig. 1.**
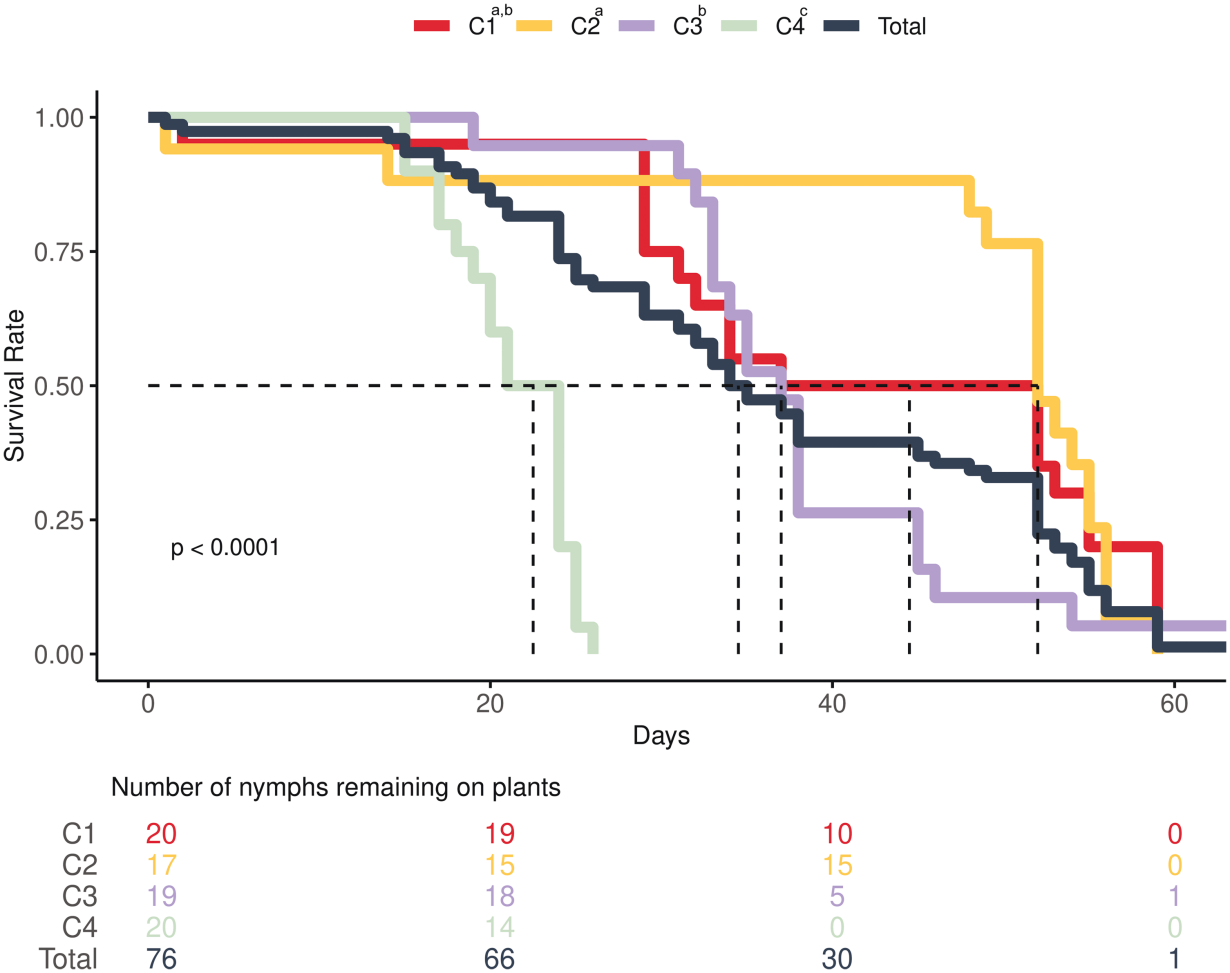
Survival curves of SLF (*Lycorma delicatula*) nymphs on each of 4 Washington navel orange plants (*Citrus sinensis*) (C1, C2, C3, and C4) (*χ*^2^ = 73.2; *df* = 3; *P* < 0.001, log-rank test). The dashed lines indicate the median survival of nymphs. Repetitions sharing the same letter are not significantly different from each other in overall survival (*α* = 0.05, log-rank test with Bonferroni correction). Below the curves, a risk table shows the number of nymphs remaining alive on each individual plant replicated at corresponding time intervals in the survival curves.

Upon arrival at UCR-I&Q, SLF egg masses were placed in an incubator (I30BLL Incubator, Percival Scientific, Inc. Perry, IA, USA) set at a constant 5 °C and 75% RH, with no light, for at least 4 wk. In preparation for experimental trials, egg masses were placed in mesh cages (W60 × H60 × D60 cm) held in a rearing room that was maintained at 25 °C and 75% RH with a 16:8 L:D cycle. Egg masses were checked daily for SLF nymph emergence. Within 24 h of hatching, twenty SLF nymphs were placed onto *C. sinensis* plants (*n* = 4 plants, for a total of 80 nymphs) held individually in mesh cages under the conditions described above. Trials began in late April 2023. Cages were checked daily for exuviae and dead nymphs. This process continued until all nymphs on each of the 4 plants died. Across 2 plants, after a thorough inspection of all plants and cages, 4 nymphs remained unaccounted for at the end of the trials. Thus, these 4 nymphs were excluded from survival analyses (see Fig. 1 below), although they were included in developmental analyses (see Table 1 below).

**Table 1.** The starting number of first-instar spotted lanternfly (*Lycorma delicatula*) nymphs and the number of nymphs that successfully developed to subsequent instars is shown for each Washington navel orange (*Citrus sinensis*) plant. The percentage of nymphs that successfully developed from the first to the second and third instars is shown in parenthesis. The average development time (days) of the first and second instars is given based on molting dates. Values sharing the same letter within the same column are not significantly different from each other (average development time of first-instar *F* = 3.8; *df* = 3; *P* < 0.05, one-way ANOVA with Tukey’s HSD; the proportion of nymphs developed to the second and third instars *α* = 0.05, Fisher’s exact test with Bonferroni correction)

Host plant	Plant ID	First-instar	Average development (days ± SD)	Second-instar	Average development (days ± SD)		Third instar
					min.	max.	
*C. sinensis*	C1	20	13.7 ± 0.7^ab^	19^a^ (95%)	14.4 ± 2.3	16.6 ± 2.4	16^a^ (80%)
	C2	20	14.0 ± 1.1^ab^	18^a^ (90%)	12.6 ± 1.5	15.6 ± 1.4	18^a^ (90%)
	C3	20	12.9 ± 1.8^a^	20^a^ (100%)	21.0 ± 8.7	27.0 ± 8.7	3^b^ (15%)
	C4	20	14.3 ± 1.6^b^	17^a^ (55%)	–	–	–^b^

The survival rates of SLF nymphs on each of the 4 *C. sinensis* plants were assessed using a Kaplan–Meier survival analysis followed by a log-rank test (Kaplan and Meier 1958, Mantel 1966). To conduct these analyses, the *survival* (Therneau 2024) and *survminer* (Kassambara et al. 2021) packages in R v4.3.3 (R Core Team 2024) were used, with the host plant as the dependent variable and nymph survival as the response variable.

The development time of each nymphal stage was calculated only for those individuals that successfully developed to the next instar. The development time of first-instar nymphs was defined by the number of days after which first-instar exuviae appeared. Differences in mean development time among first-instar nymphs on plants were analyzed in R using one-way ANOVA with Tukey’s HSD used for means separation (*α* = 0.05) (Tukey 1949). Since it was not possible to individually track nymphs on trees during experiments, the development time of second-instar nymphs was estimated by calculating an average minimum and maximum time in the stadium for each nymph. These minimum and maximum estimates were based on the difference in days between the appearance of first- and second-instar exuviae. Subsequently, the average minimum and maximum development times of nymphs were calculated for each individual plant (see Table 1 below). Differences in the proportions of nymphs reaching the second and third instar in each repetition were assessed in R using Fisher’s exact test with Bonferroni correction to account for pairwise comparisons (*α* = 0.05) (Fisher 1922).

## Results and Discussion

Under the experimental conditions of this study, the median and maximum survival time of SLF nymphs reared on *C. sinensis* were 34.5 and 66 days, respectively, with differences in overall survival among replicates (*χ*^2^ = 73.2; *df* = 3; *P* < 0.001, log-rank test) (Fig. 1). Overall, the proportion of nymphs that reached the second and third instar was 92.5% and 47.4%, respectively. There was no significant difference in the proportion of nymphs reaching the second-instar among host plants, but there was a significant difference in the proportion reaching the third instar with respect to host plant replicate (*α* = 0.05, Fisher’s exact test with Bonferroni correction) (Table 1). The mean time to develop across replicates from first to second-instar was 13.7 ± 1.43 days, with a significant difference between the replicates showing the minimum (12.9 ± 1.8) and maximum (14.3 ± 1.6) values (*F* = 3.8; *df* = 3; *P* < 0.05) (Table 1).

The significant differences observed among replicates were attributed to the health of the plants. For example, replicate C4 developed a soft-scale infestation, leading to its premature decline and death. This replicate subsequently exhibited significantly lower SLF nymph performance after the emergence of second-instar nymphs (Fig. 1; Table 1). Furthermore, due to space restrictions in UCR-I&Q, experimental trees were not replaced during the experiment, unlike some other rearing studies (e.g., Nixon et al. 2022, Elsensohn et al. 2023a,b). As a result, honeydew produced by SLF nymphs accumulated on the plants, potentially impacting the health of the plants and the overall performance of SLF nymphs. These experimental shortcomings introduced bias, which might have negatively affected the results, possibly leading to an underestimation of the suitability of *C. sinensis* for SLF development.

Regardless of these shortcomings, the mean development time of first-instar nymphs on *C. sinensis* was about 25% faster than on preferred hosts (e.g., *A. altissima* and *Vitis* spp., alone or when paired with other plants) in similarly designed experiments (Elsensohn et al. 2023a, b, Madalinska and Nielsen 2024). The proportion of nymphs reaching the third instar also aligned with results from these experiments (Elsensohn et al. 2023a,b, Madalinska and Nielsen 2024) (Table 1). Although results from this work are not directly comparable to these other studies, the findings reported here support the hypothesis that *C. sinensis* might be a high-quality host for the SLF. Although no SLF nymphs developed beyond the third instar, this outcome was likely due to excessive honeydew accumulation on host plants and the limitations of our experimental design.

Results from this quarantine-based study have provided evidence that *C. sinensis* can support the survival of SLF nymphs for multiple weeks and over multiple developmental stages. This is of particular significance because areas identified as being at high risk of future SLF invasion overlap with regions where citrus is a widespread and economically important crop (e.g., Brazil, California (USA), Italy, Spain) (Wakie et al. 2020, Zhong and Nicolosi 2020, Huron et al. 2022, Jones et al. 2022). The identification of *C. sinensis* as a possible suitable host for SLF broadens the range of economically important plants potentially at risk as SLF continues to expand its range (Huron et al. 2022). This may be of particular concern for the state of California (USA), which accounts for 79% of total US citrus production, with an estimated total value of approximately $2.2 billion ($US) (packinghouse-door equivalent) (2022–2023, USDA 2023) and where the invasion of SLF has been predicted to occur within the next 10 years (Jones et al. 2022). Furthermore, although it is uncertain if the citrus industry is threatened by SLF, the presence of citrus plants might facilitate the spread of this pest as it invades new areas, thereby increasing risks to other vulnerable crops such as grapevines (Urban et al. 2021, Huron et al. 2022).

In conclusion, the results reported here are, to the best of our knowledge, the first records of SLF nymphs surviving and developing on any *Citrus* species. Therefore, it is recommended that additional studies be undertaken to fully determine the suitability *Citrus* sp. as hosts for SLF so that potential negative impacts can be better understood and prepared for.
